# Comparison of Combined Femoral Nerve Block and Spinal Anesthesia With Lumbar Plexus Block for Postoperative Analgesia in Intertrochanteric Fracture Surgery

**DOI:** 10.5812/aapm.4526

**Published:** 2012-07-10

**Authors:** Hamid Reza Amiri, Saeid Safari, Jalil Makarem, Mojgan Rahimi, Behnaz Jahanshahi

**Affiliations:** 1 Department of Anesthesiology and Intensive Care, Imam Khomeini Hospital, Tehran University of Medical Sciences (TUMS), Tehran, Iran; 2 Department of Anesthesiology, Rasoul Akram Medical Center, Iran University of Medical Sciences (IUMS), Tehran, Iran

**Keywords:** Femoral Nerve, Anesthesia, Spinal, Lumbosacral Plexus, Analgesia, Hip Fractures

## Abstract

**Background:**

Hip fracture–related pain both before and after surgery is generally reported as severe by most patients. Various regional pain control modalities have been described in order to reduce pain in these patients.

**Objectives:**

Because of the challenges of lumbar plexus block (LPB) and the fact that the effect of combined femoral nerve block/spinal anesthesia in controlling pain after orthopedic surgeries has not been investigated, in this study, we compared the feasibility and efficacy of the 2 techniques in the perioperative management of proximal hip fractures.

**Patients and Methods:**

The study included 32 patients with femoral intertrochanteric fracture who were randomly divided into the following 2 groups of 16 patients each: combined femoral nerve block/spinal anesthesia group (group I) and LPB group (group II). Patients in group I received 0.17% bupivacaine with 0.7% lidocaine, 20–25 mL for femoral nerve block and bupivacaine 0.5% plus 0.5 mL pethidine (25 mg) for spinal block and patients in group II received 0.17% bupivacaine with 0.7% lidocaine, 30–35 mL.

**Results:**

The time for performing the block (12.2 ± 3.3 *vs*. 4.93 ± 1.6 min, *P* = 0.001) and achieving the block (7.7 ± 0.9 *vs*. 2.4 ± 1.0 min, *P* = 0.001) were significantly longer in the combined femoral nerve block/spinal anesthesia group than in the LPB group. Duration of analgesia in the combined femoral nerve block/spinal anesthesia group was longer than that in the LPB group, but the difference was not significant (17 ± 7.3 *vs*. 16.5 ± 8.5 h, *P* = 0.873). There were no significant differences in hemodynamic parameters regarding the method of anesthesia in the 2 groups.

**Conclusions:**

This study confirms that the combination of femoral nerve block with spinal anesthesia is safe and comparable with LPB and can provide more effective anesthesia and longer lasting analgesia for intertrochanteric surgery.

## 1. Background

Proximal femoral fracture (hip fracture) involves a fracture of the femur in the area of bone immediately distal to the articular cartilage of the hip, to a level of about 5 centimeters below the lower border of the lesser trochanter. The majority of these fractures occur in an elderly population, and patients with hip fractures frequently have various co-morbidities attributable to the normal process of ageing (1, 2). Due to increasing the age of population in most countries, it seems reasonable that we encounter more cases of hip fractures now than in previous decades. Surgical repair is the method of choice to treat such fractures. As a result, surgery for hip fracture represents one of the most common emergency orthopedic procedures performed (3–6). Hip fracture-related pain both before and after surgery is usually reported as severe by most patients. Those who report more severe pain after surgery have longer hospital stays and greater delays before mobilization (1). There is a tendency among anesthesiologists to provide pain relief in elderly patients with substantial amounts of analgesics post-operatively. This practice may pose risks in this age group, mainly due to the concurrent co-morbidities. The opiate drugs that are administered commonly for pain relief have complications including depression of central respiratory centers, drowsiness, hypotension, and mental confusion. On the other hand, anti-inflammatory agents such as non-steroidal anti-inflammatory drugs (NSAIDs) may increase the risk of bleeding and gastrointestinal hemorrhage, and may adversely affect renal function in susceptible patients. Thus, in order to reduce pain in these patients, various regional pain control modalities have been described. Regional nerve blocks can include the lateral cutaneous nerve of the thigh, the subcostal nerve, the femoral nerve, the sciatic nerve, triple nerve block (femoral, obturator and sciatic nerves), psoas (lumbar plexus) block, or continuous epidural block (2).

## 2. Objectives

In this study, we compared the feasibility and efficacy of lumbar plexus block (LPB) to combined femoral nerve block/spinal anesthesia in the perioperative management of proximal hip fractures.

## 3. Patients and Methods

This study was performed as a single-blind randomized clinical trial after receiving institutional review board approval and informed consent from the patients. The participants were 32 patients with femoral intertrochanteric fracture who presented to Imam Khomeini Medical Center. Inclusion criteria were age >18 years, ASA class I–III, and weight > 50 kg. Exclusion criteria were multiple fractures, peripheral neuropathy, bleeding disorders, mental disorders, communication failure, allergy to local anesthetics, opium abuse, and use of analgesics for premedication. The patients were allocated by computer-generated random numbers into 2 groups of 16 patients each: combined femoral nerve block/spinal anesthesia (group I) *vs.* LPB (group II). The random allocation sequence was concealed in sealed opaque envelopes until a group was assigned. All patients were monitored with non-invasive blood pressure measurements, electrocardiography, pulse oximetry, and qualitative ETco2. An infusion of lactated Ringer’s solution was given, and all patients were supplied with oxygen (6 L/min) via a face mask. Midazolam (0.025 mg/kg) was used as pre-medication. Patients assigned to the LPB group were administered a single injection using the approach described by Winnie *et a1.* (6). The LP was localized by inducing contractions of the quadriceps femoris using a nerve stimulator (Poly medic, USA) delivering 0.3–0.7 mA impulses of 0.1 ms at 1 Hz, linked to a 23 gauge, 120-mm Teflon coated short bevel sterile needle. After repeated negative aspirations, 30–35 mL bupivacaine, 0.17% with 0.7% lidocaine was injected. Patients in the combined femoral nerve block/spinal anesthesia group underwent femoral nerve block guided by a peripheral nerve stimulator. An insulated 50 mm 23 G needle was introduced 1 cm lateral to the femoral artery and just below the inguinal ligament. When a current 0.3–0.7 mA elicited a quadriceps contraction, bupivacaine 0.17% with 0.7% lidocaine, 20–25 mL was injected incrementally after multiple negative aspirations. The patient was then turned into the lateral position with the fracture site up. The spinal block was performed by either a midline or paramedian approach at the L2/3 or L3/4 level with 25 G Quincke needle and 1.5 mL of hyperbaric bupivacaine 0.5% with 0.5 mL pethidine (25 mg). During the first 24 h post-operatively, the patients were instructed to inform the ward nurses when they were suffering from pain. If any patient in either group reported pain scores > 3 while changing position, 0.05 mg/kg intramuscular morphine sulfate was administered until the pain score decreased to ≤ 3. The individual who measured the pain scores of the patients was blinded to the study. Length of time for performing the blocks (time between beginning of prep/drape and withdrawal of the block needle), time to achieving block (time between withdrawing the block needle and reduction of patient’s pain), operation time (min), time to the first request for analgesia (h) were compared between the 2 groups. The main postoperative anesthesia-related complications, including nausea, vomiting, pruritus, urinary retention, drowsiness, and respiratory depression were measured. Parametric variables were described as mean ± SD; qualitative variables were described as number (percentage) and as median and range. Student’s *t* test, chi-square test or Fisher exact test, or Mann-Whitney *U* test was used as appropriate to compare the 2 groups. *P* < 0.05 was considered statistically significant. Data were analyzed using an SPSS 13.0 software package.

## 4. Results

Demographics according to ASA physical status, age, sex, and weight were not significantly different between the treatment groups (*Table 1*). The time for performing the block was significantly shorter in LPB (*P* = 0.001). The time to achieving the block was significantly longer in the combined femoral nerve block/spinal anesthesia (*P* = 0.001) group, but times to the first request for analgesia were comparable between the groups (*P* > 0.05) (*Table 2*). The satisfaction rating regarding the method of anesthesia was 8.9 ± 1 for LPB *vs.* 8.7 ± 1.2 for femoral nerve block/spinal anesthesia among the surgeons and 8.1 ± 1.5 *vs.* 7.9 ± 1.8 among the patients (*P* > 0.05). The hemodynamic parameters were compared between 2 groups, and the results are listed in *Table 3*. The main postoperative anesthesia-related complications such as nausea, vomiting, pruritus, urinary retention, drowsiness, and respiratory depression were compared between the groups (*Table 4*). Occurrence of nausea was significantly higher in the femoral nerve block/spinal anesthesia group (7 patients) than in the LPB group (1 patient).

**Table 1:** Demographic Characteristics of Patients in Lumbar Plexus Block and Combined Femoral Nerve Block/Spinal Anesthesia Groups

	**Lumbar Plexus Block, n = 16**	**Femoral Nerve Block Plus Spinal Anesthesia, n = 16**	***P* value**
Age, y, mean ± SD	64.4 ± 14.7	65.6 ± 12.6	0.81
Sex, male/female	8/8	7/9	0.99
ASA physical state, No. (%)			
I	5 (31.25)	5 (31.3)	0.59
II	1 (6.25)	0 (0)	
III	10 (62.5)	11 (68.8)	
BMI ^a^, kg/m^2^, mean ± SD	21.6 ± 5.9	22.1 ± 6.1	0.46
		

a Abbreviation: BMI, body mass index

**Table 2:** Comparison of Anesthesia Parameters Between the 2 Groups

	**Lumbar Plexus Block, n = 16**	**Femoral Nerve Block + Spinal Anesthesia, n = 16**	***P* value**
Time of performing block, min, mean ± SD	4.93 ± 1.6	12.2 ± 3.3	0.001
Time of achieving block, min, mean ± SD	2.4 ± 1.0	7.7 ± 0.9	0.001
Operation time, min, mean ± SD	158.8 ± 38.9	155.3 ± 39.1	0.677
Time to the first demand for analgesia, h, mean ± SD	16.5 ± 8.5	17 ± 7.3	0.873

**Table 3:** Comparison of Hemodynamic Parameters Before and After Lumbar Plexus or Femoral-Neuroaxial Blockade

	**Lumbar Plexus Block, n = 16**	**Femoral Nerve Block + Spinal Anesthesia, n = 16**	***P* value**
Pre-systolic blood pressure, mean ± SD	132.81 ± 20.6	134.93 ± 17.2	0.8
Pre-diastolic blood pressure, mean ± SD	57.31 ± 29.3	54.5 ± 10.2	0.9
Pre-heart rate, mean ± SD	88.6 ± 13.5	86.4 ± 6.9	0.8
Post-systolic blood pressure, mean ± SD	127.3 ± 19.1	113.1 ± 16.5	0.5
Post-diastolic blood pressure, mean ± SD	67.9 ± 9.7	66.8 ± 7.5	0.6
Post-heart rate, mean ± SD	82.7 ± 14.2	84.6 ± 8.4	0.7

**Table 4:** Comparison of Complications in the 2 Groups

	**Lumbar Plexus Block, n = 16**	**Femoral Nerve Block + Spinal Anesthesia, n = 16**	***P* value ^a^**
Nausea, No.	1	7	0.037
Vomiting, No.	0	3	0.23
Pruritus, No.	1	5	0.17
Urinary retention, No.	0	2	0.48
Drowsiness, No.	2	3	0.99

a Fisher’s exact test was used

## 5. Discussion

Intraoperative anesthesia management and postoperative pain control with 2 regional anesthesia methods were compared in this study. Although major hemodynamic changes and fluid shifts due to extensive sympathetic block, especially in patients with cardiovascular compromise, are of great concern, spinal anesthesia is considered as a primary method of anesthesia in lower extremity surgeries. LPB may be accompanied by less sympathetic involvement because of the unilateral approach and somatic dominant effect. In this study, hemodynamic changes in lateral position after LPB were comparable to those of spinal anesthesia, indicating that LPB may be considered safe in patients with cardiovascular compromise. On the other hand, during surgery, sufficient muscle paralysis is of great importance in the reduction of fractures, particularly in densely muscular regions, and may be evaluated by surgeons as surgeon satisfaction. This study indicates that in elderly patients, LPB provided sufficient relaxation comparable to spinal anesthesia. The duration of analgesia with peripheral nerve blocks is longer than spinal anesthesia. In this study, combining a femoral nerve block with spinal anesthesia provided better pain free positioning for the spinal anesthesia procedure and yielded pain control comparable to the LPB. A recent meta-analysis regarding anesthesia for major orthopedic surgical procedures of the knee reported that blocking of peripheral nerves in the lower extremity resulted in an acceptable rate of postoperative analgesia. This level of analgesia was comparable with epidural infusion and had fewer side effects such as hypotension, urinary retention, nausea, and itch (7). Several reports during recent years have compared femoral nerve block with LPB for postoperative pain treatment after lower limb surgery, and many investigators have noted that continuous LPB has similar efficacy to continuous femoral block, either with or without sciatic nerve block (8). Compared with an inguinal paravascular approach, LPB was shown to be an effective approach to ensure a good anesthesia to all the branches of the plexus (femoral nerve, obturator nerve, femorocutaneous nerve). Marino *et al.* reported that femoral blocks and continuous LP significantly reduced the need for opioids. Continuous LPB was a more effective analgesic intervention than continuous femoral block alone following primary unilateral total hip arthroplasty (12).

As a limitation in our study, the sample size was not large. Nevertheless, considering that the patients with hip fracture may have greater numbers of comorbities and the potential for risk from severe cardiovascular changes during surgery, the combination femoral nerve block and spinal anesthesia can be safely recommended for pain management in high risk patients.

In conclusion, for anesthesia management of hip fractures in elderly patients, a single shot LPB provided satisfactory intraoperative conditions and considerable postoperative pain control. LPB can be considered as a primary option, is less time consuming, and requires only 1 skin puncture site. However, as the proficiency and expertise required in LPB may matter, if spinal anesthesia is scheduled, providing a femoral nerve block prior to the subdural injection may produce better post-operative analgesia.
